# Bartha-K61 vaccine protects nursery pigs against challenge with novel european and asian strains of suid herpesvirus 1

**DOI:** 10.1186/s13567-022-01063-8

**Published:** 2022-06-22

**Authors:** Konstantinos V. Papageorgiou, Margarita Michailidou, Ioannis Grivas, Evanthia Petridou, Efthymia Stamelou, Konstantinos Efraimidis, Lei Chen, Trevor W. Drew, Spyridon K. Kritas

**Affiliations:** 1 Department of Microbiology and Infectious Diseases, Faculty of Veterinary Medicine, School of Health Sciences, Aristotle University of Thessaloniki (AUT), 54124 Thessaloniki, Greece; 2 Laboratory of Anatomy Histology and Embryology, Faculty of Veterinary Medicine, School of Health Sciences, Aristotle University of Thessaloniki (AUT), 54124 Thessaloniki, Greece; 3 College of Veterinary Medicine, Yangzhou University, Yangzhou, China; 4 CSIRO-Australian Centre for Disease Preparedness, 5 Portarlington Road, Geelong, VIC3200 Australia

**Keywords:** Pseudorabies, Aujeszky’s disease, Bartha-K61 vaccine, pig, variant

## Abstract

The present study investigates the pathogenicity of two recently isolated strains of Suid herpesvirus 1 (SuHV1), the Greek strain Hercules and the Chinese strain HeN1, in unvaccinated pigs and in pigs vaccinated with a Bartha-K61 strain. In an experiment performed in negative pressure kiosks (isolators), 45-day old seronegative pigs previously oronasally /intramuscularly vaccinated with the Bartha-K61 vaccine strain, along with unvaccinated controls, were challenged either with the Hercules strain or the HeN1 strain of SuHV1. All animals were observed daily for clinical signs and body temperature and nasal swabs, faeces, blood and bodyweight were collected up to a maximum period of 20 days post-challenge (dpc). The results showed that, in the unvaccinated pigs, HeN1 strain was more virulent than the Hercules strain, with increased mortality, shorter time to death and higher group clinical score (*p* < 0.05). However, after vaccination with the Bartha-K61 vaccine, there was a drastic reduction in morbidity, mortality, bodyweight loss and virus excretion to almost a similar extent in both strains (*p* < 0.05). No significant differences were seen among the pigs of the two vaccinated groups compared to unvaccinated unchallenged controls, except a slight elevation in body temperature and in clinical score in the HeN1 vaccinees at 2 and 3 dpc, while bodyweight gain was similar to that of the negative control pigs. Our study showed that despite differences in virulence, the standard vaccination scheme with the Bartha-K61 strain could equally protect nursery pigs against both the European and Chinese strains.

## Introduction

Aujeszky’s Disease (AD) or pseudorabies, caused by Suid Herpesvirus 1 (SuHV1), is an acute, frequently fatal disease, that primarily affects pigs and incidentally other domestic and wild animals [1, 2]. As pig production has grown since the 1960’s, SuHV1 has emerged as an important global pathogen. Today, although it has been eradicated from several countries, the disease mostly remains endemic in many regions of the world [2].

The manifestation of the disease varies depending on the age of infected pigs, the virulence of the infecting strain, and whether or not previous exposure had occurred, either of the animal itself, or, for neonates, the immune status of the dam [3]. In non-immune pigs, neonates usually die demonstrating typical neurological signs (e.g. trembling, weak hindlimbs and loss of coordination), while weaners (3–9 weeks of age) show clinical signs similar to those observed in suckling pigs but less severe. In growers and fatteners, SuHV1 infection is usually mild and it is characterized mainly by the development of respiratory signs, whilst in sows reproductive disorders are seen [2, 3]. Although one serotype of the virus is known, some strains seem to exhibit increased virulence in seronegative pigs [4–6].

Live attenuated or inactivated vaccines lacking gE glycoprotein are successfully used to control AD [7–9]. Most attenuated vaccines use the Bartha-K61 strain [10] and many countries of Europe have eradicated the disease by using vaccines incorporating this strain. In countries without an intensive eradication programme, optional vaccinations have reduced clinical disease but do not eliminate the virus [5, 10]. In Greece, although vaccination against AD is widely practiced, almost 30% of farms have been demonstrated to be SuHV1 positive [11]. It has never been investigated whether the ineffective protection in such farms is due to the vaccine strain itself, or due to inconsistent application of vaccination.

Since late 2011, novel strains have emerged in China on farms where Bartha-K61 vaccines were used [12, 13]. These recently emerged strains have been classified as a new genotype II [14], being distinct from strains known up to that time, which were assigned to genotype I. In a limited number of laboratory studies, the Bartha-K61 vaccines were claimed to show either reduced efficacy [15–18] or similar efficacy [19–21] against Chinese genotype II field strains, compared to other Chinese vaccine candidates.

The purpose of the current study was to compare the pathogenicity of two recently isolated field strains, one of European genotype I and the other of Chinese genotype II origin, on seronegative pigs and to test the efficacy of a well-known attenuated Bartha-K61 vaccine strain against these two strains.

## Materials and methods

### Viruses and commercial vaccine

Two virulent field strains of SuHV1 both isolated from brain tissue homogenates from pigs with neurological problems were used: a) the European Greek strain (Hercules) isolated ιn 2010 on a farm in Greece, with neurological problems in neonatal pigs and farm pets [22], and b) the Chinese strain (HeN1) isolated ιn 2012 from a farm where vaccination with a Bartha vaccine had previously been carried out [12]. Both strains were propagated after 2 το 3 passages in PK-15 cell culture and virus stock harvested by freeze/ thawing and clarified by centrifugation at 1000 *g* for 10 min at 4 °C. Virus stocks were tested for adventitious agents and neither mycoplasma [23] nor known viruses were detected, applying a pan viral microarray [24].

Hercules strain had been characterised by whole genome sequencing [22] and according to the recent proposal for SuHV1 genotyping [14] belongs to genotype I including all known non-Chinese strains. In contrast, HeN1 belongs to putative genotype II, which includes recently emerged Chinese strains [14]. Based on distance calculations, these two SuHV1 strains had been designated as SuHV-1.2 (main European/American) and SuHV-1.1 (Chinese), respectively [25].

The vaccine used in this study was the Ingelvac® Aujeszky MLV (Lot No 1951138A Boehringer Ingelheim, Germany), an attenuated (live) vaccine based on Bartha-K61 strain of SuHV1.

### Animals and housing

Pigs were obtained from a farrow-to-finish commercial farm of 300 sows that have been serologically tested PRRSV-free, PCV2-free and SuHV1-free, and where no vaccinations against any of these diseases had been performed.

**Figure 1:**
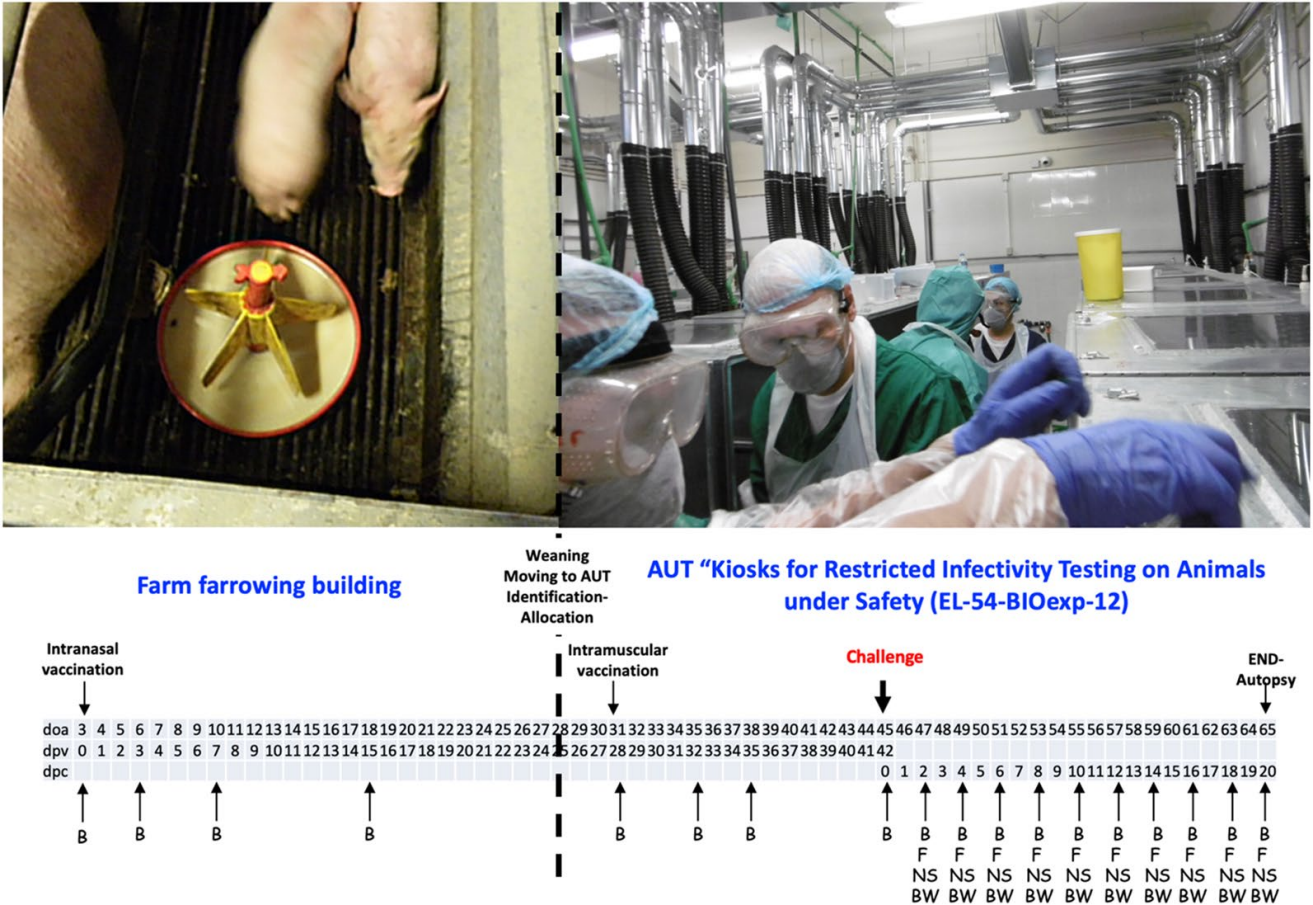
**Graphic display of the experimental study design including a timeline of vaccination, challenge, sampling (B-blood, NS-nasal, F-faecal swabs, BW-bodyweight) timepoints. **Clinical signs and body temperature were recoded daily. Tissues (nasal mucosa, brain) were collected during pig death or final autopsy (doa = days of age; dpv = days post-vaccination; dpc = days post-challenge).

This study was initiated on the farm of origin, in an isolation room, where piglets received the first vaccine dose. Subsequently piglets were removed in a specialist animal facility at Aristotle University of Thessaloniki (AUT) “Kiosks for Restricted Infectivity Testing on Animals under Safety (EL-54-BIOexp-12)” (license No 290287/2442-29.09.2014) fulfilling animal biosafety level-3 requirements, where the second vaccination and challenge study was performed. The dimensions of each kiosk were L80 cm xW80 cm xH140 cm (Figure 1).

At weaning at 28 days of age (doa), the piglets were moved to AUT for revaccination and challenge (Figure 1). On arrival, all piglets were identified by a thermal microchip (BioThermo Lifechip, Destron Fearing, St Paul, Minnesota, USA), subcutaneously implanted in the neck area, and randomised among and within groups, according to litter of origin and to gender. All pigs were supplied with pre-weighed feed and water ad libitum during the study. As each pig was housed individually and was completely autonomous from the others, each piglet represented a single experimental unit (replicate).

### Vaccination and challenge procedures

At the farm of origin, two pregnant dams were both moved to an isolated room. Three days after farrowing, selected piglets of their litters were intranasally vaccinated with 2 mL of the vaccine (1 mL or 10^4.9^ TCID_50_ per nostril), as per the manufacturer’s protocol and left with their dams (Figure 1).

At weaning, at 28 doa, 10 vaccinated piglets from these two selected dams and 14 unvaccinated piglets from the regular farrowing house of the farm were moved to AUT and housed in individual experimental kiosks to acclimatise (Figure 1).

At 31 doa (4 weeks after the first vaccination), the once-vaccinated pigs received intramuscularly a second booster dose (2 mL) of the vaccine as per manufacturer’s protocol (Figure 1).

Challenge of the recipient pigs (vaccinated or not) was performed at 45 doa—42 days after the first vaccination and 14 days after the second vaccination (Figure 1). The challenge strain (Hercules or HeN1) was oronasally inoculated in a total volume of 4 mL containing 10^7.5^ TCID_50_ /mL (e.g. 10^8.1^ TCID_50_ /pig). For challenge, the sedated pigs were placed on sternal recumbency and by using a 4 cm-long catheter, a 1 mL suspension was administered in each nostril and a 2 mL suspension in the oral cavity. The pig were kept in this position for 5 min. Of the remaining inoculum, a back-titration was performed (as demonstrated to “Virus quantitation” section) to assure the correct virus titre.

### Experimental design

Twenty-four 45-day old pigs were allocated in 5 groups: G1 with unvaccinated and unchallenged control pigs (*n* = 4); G2 with unvaccinated pigs and oronasally challenged with the Hercules strain (*n* = 5); G3 with unvaccinated pigs and oronasally challenged with the HeN1 strain (*n* = 5); G4 previously vaccinated pigs and oronasally challenged as G2 pigs (*n* = 5); G5 previously vaccinated pigs and oronasally challenged as G3 pigs (*n* = 5).

Blood samples were obtained at doa 3 (primary vaccination), 6, 10, 18, 31 (booster vaccination), 35, 38 and 45 (challenge) from all pigs. After challenge (doa 45) all animals were daily observed for clinical signs and body temperature, while every other day nasal swabs, faeces, blood and bodyweight (BW) were collected up to a maximum period of 20 days post-challenge (dpc), e.g. the day of euthanasia (Figure 1).

### Clinical signs and sample collection

Pigs were observed three times daily (morning/noon/evening) for clinical signs. The following scale was used to grade daily signs of each pig:

- Rectal temperature score: (0) normal (≤ 39.5 °C), (1) raised (39.6–40.2 °C), (2) fever (40.3–40.9 °C), (3) high fever (≥ 41 °C),
- Appetite: (0) Greedy, hungry, trough empty, (1) Eats when fed, trough almost empty, almost no leftovers, (2) Does not eat when fed but tastes food, food only partially eaten, (3) Does not eat at all, shows no interest for food, trough still full, nothing eaten.
- Liveliness: (0) Attentive (curious, stands up immediately), (1) Slightly reduced (Stands up hesitantly but without help), (2) Tired, gets up only when forced to, lies down again, (3) Dormant, will not stand up
- Gait-Stance: (0) Well coordinated movements, (1) Hesitant walking or slow, awkward gait, (2) Distinct ataxia/lameness, able to walk, (3) Significant lameness, unable to walk.
- Neurological signs: (0) Normal movements of head and body, (1) Mild neurological signs (e.g. trembling, jerky movements and poor coordination) for short periods, animal being conscious, (2) More severe neurological signs (e.g. circling, head-pressing, head-tilt) with raised animal losing its consciousness, (3) Recumbency, loss of consciousness, coma, paralysis, paddling, convulsion/ seizures.
- Other signs: (0) None, (1) hyperaemia (of ears, nose, belly and legs), rhinitis, sneezing, (2) dyspnoea, emaciation, (3) death.

The pig daily clinical score (PDCS) was the sum of the sign grades of each day for each pig.

Body temperature (as adjusted to rectal temperature) was collected once daily (at 8 am) using Global Pocket Reader Plus scanner (Destron Fearing, St Paul, Minnesota, USA) according to the manufacturer’s instructions before placing the preweighed amount of feed in the feeder.

At the sample collection dates, and after clinical observation and body temperature collection, pigs were sedated by a combination of 2–2.5 mg azaperone /kg bodyweight (Stresnil, Elanco) and 10–15 mg Ketamine HCl /kg bodyweight (Ketamidor, Richter Pharma AG) with deep intramuscular injection. The animal handlers changed oversuits and hairnets, respiratory masks, goggles and gloves between groups. Handling order started with control groups, then Hercules (first vaccinated and then unvaccinated) and then HeN1 (first vaccinated and then unvaccinated).

All sampling and measurements were carried out within individual kiosks. From each sedated pig, blood was collected by jugular venipuncture, followed by collection of nasal mucus using preweighed swabs, and fecal material. Each pig was then weighed and the relative BW increase compared to the BW at the date of challenge was calculated for each pig.

Sick animals showing severe neurological signs leading to coma (neurological score 3) or/and with PDCS higher than 10 were euthanized immediately. The heads of these animals, also those that were found dead, were stored at −80 °C. At the end of the experiment at 20 dpc, all remaining animals were euthanized and nasal mucosa and brain samples were taken, comprising pooled pons, medulla and cerebral cortex. Similar samples were obtained from the stored material.

### Serum neutralization (SN)

Sera were inactivated for 30 min at 56 °C and were examined for SN antibodies by preparing twofold dilutions in a 96-well plate. The dilutions were mixed with an equal volume of SuHV1 (Ka strain belonging to SuHV-1.2 genotype) [26] containing approximately 100 TCID_50_. After 1-h incubation at 37 °C, approximately 25 000 cells of the PK15 cell line were seeded in each well. SN titres were calculated by the Reed-Muench method as the reciprocal of the highest dilution which inhibited the viral cytopathic effect in 50% of the cell cultures after 5 days [26].

### ELISA

Sera were examined for the presence of gE and gB antibodies by using PRV/gB and PRV/gE competitive antibody ELISA kits as per manufacturers instructions (IDEXX Laboratories, Westbrook, ME, USA), respectively.

### Virus quantitation

Twenty percent (20% w/v) suspensions were made from tissues in phosphate buffered saline (PBS). Nasal and fecal swabs were weighed and 1 mL of PBS was added (supplemented with 10% fetal calf serum, 200 IU/mL penicillin and 100 µg/mL streptomycin in fecal swabs) and shaken for 60 min at 4 °C.

The suspensions were centrifuged at 1000 *g* for 20 min at 4 °C. Subsequently, 0.1 mL of supernatant was inoculated on each of 4 wells of confluent PK-15 cells on a 96-well plate. The cell cultures were observed during 5 days for cytopathic effect. For virus quantitation,tenfold dilutions of the original suspensions were titrated on the cells and the end point titres were calculated by the Reed-Muench method.

### Statistical analysis

Since all animals were kept in individual isolators, the experimental unit for statistical purposes was defined as the individual pig. The data were analyzed using SPSS software (version 25.0, provided by the AUT). Various post-hoc power analyses were conducted for the computation of the achieved power of the statistical analysis given alpha (5%), sample size and effect size using body temperature and bodyweight data from each experimental group as one of the primary output of the study using G*Power software (free version, Heinrich Heine-University). For continuous quantitative variables (rectal temperatures, bodyweight increase, SN titres), a repeated measures Analysis of Variance (ANOVA) was used for the statistical comparisons between groups except for nasal excretion for which one-way ANOVA within each day was opted instead. For ordinal scale variables (i.e. clinical scoring) a non-parametric Kruskal–Wallis ANOVA was used instead. Post-hoc multiple comparisons were performed after adjusting the possibility of achieving a type I error, by use of the Bonferroni correction. Level of significance was set at 5% (*P* ≤ 0.05).

## Results

### Clinical signs

**Figure 2:**
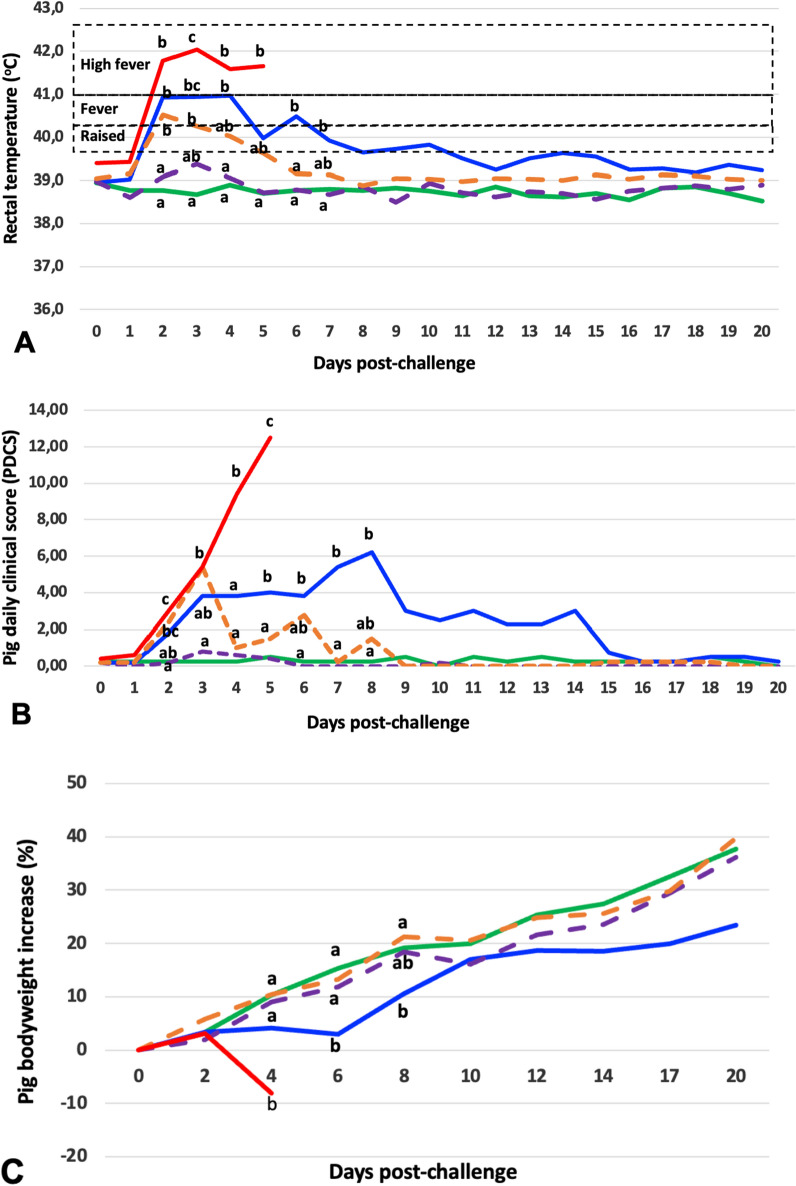
**Clinical signs of unvaccinated or Bartha-K61 vaccinated pigs challenged with Hercules and HeN1 strains of SuHV1.** **A** Rectal temperatures, **B**. Pig Daily Clinical Scores, C. Bodyweight increase. G1-Unvaccinated and unchallenged control pigs (green line; *n* = 4); G2-Unvaccinated and Hercules-challenged pigs (blue line; *n* = 5); G3-Unvaccinated and HeN1-challenged pigs (red line; *n* = 5); G4-Vaccinated and Hercules-challenged pigs (dashed violet line; *n* = 5); G5-Vaccinated and HeN1-challenged pigs (dashed orange line; *n* = 5). Different letters at the same day post-challenge show significant difference (*p* < 0.05).

None of the control (G1) pigs showed substantial elevation of rectal temperature (Figure 2A) or any other clinical signs (Figure 2B).

In unvaccinated Hercules infected (G2) pigs, anorexia and high fever (average 41 °C) from 2 to 4 dpc was seen. Subsequently, a slight fever reduction but still above 40 °C was observed up to 20 dpc (Figure 2A). One pig had developed moderate neurological signs such as delayed coordination and head tilt (grade 2) at 8 dpc and found dead the next day, while another G2 pig had developed head tilt from 13 dpc until 20 dpc without any other significant signs. The remaining G2 pigs had shown transient clinical signs. PDCS of G2 pigs was significantly increased from 5 dpc up to 8 dpc (*p* < 0.05) (Figure 2B).

In all unvaccinated HeN1 infected (G3) pigs, anorexia and high fever (average 41.6 °C) had been observed from 1 dpc to their euthanasia or death. One of the pigs displayed neurological signs such as paddling movements in recumbent position, while the rest of the G3 pigs died after showing the previous day only mild to moderate neurological signs (grade 1 or 2) when standing, such as depression, sniffing, chewing or/and hypersalivation. All pigs in the G3 Group died or were euthanased in an average of 5.4 dpc, with the neurological signs developing more quickly in G3 pigs, compared to the neurologically affected G2 pigs (Table 1). For G3 pigs, PDCS was significantly higher from 2 dpc up to their euthanasia or death when compared to G1, but also compared to the G2 pigs (Figure 2B) (*p* < 0.05).

**Table 1:** **Clinical characteristics of 45-day old pigs non-vaccinated or vaccinated with the Bartha strain of SuHV1 and subsequently challenged with the Hercules or HeN1 strain of SuHV1**.

	n =	Development of high fever (≥ 41 °C)		Development of neurological signs (score 2 +)		Survival			Virus titre [log_10_/g tissue]			
		Nr pigs	Avg time (days)	Nr pigs	Avg time (days)	Dead pigs	Mortality (%)	Avg time to death (days)	In dead pig		At 20 dpc^a^	
									Nasal mucosa	Brain	Nasal mucosa	Brain
Group												
G1-control	4	0	0	0	0	0	0	–	–	–	0.0	0.0
G2-Hercules	5	4	2	2	9.5	1	20.0	9	4.1	3.5	2.1 (3/4)^b^	1.3 (1/4)
G3-HeN1	5	5	2	2	4	5	100.0	5.4	4.5	4.6		
G4-VacHercules	5	0	0	0	0	0	0	–	–		1.8 (3/5)	0.0 (0/5)
G5-VacHeN1	5	1	2	0	0	0	0	–	–	–	2.4 (2/5)	0.0 (0/5)

^a^dpc = days post-challenge.

^b^positive pigs/total alive pigs.

The vaccinated Hercules-challenged pigs (G4) did not show substantial elevation of rectal temperature or any clinical signs at any time (Figure 2A) (Table 1).

In the vaccinated HeN1-challenged pig (G5), one pig developed high fever at 2 to 3 dpc without any other sign (Figure 2A). Four of the pigs of the G5 group had shown mild transient signs (fever, slight depression) until 8 dpc (*p* > 0.05). PDCS was only raised at 2 and 3 dpc (Figure 2B) (*p* < 0.05).

As presented in Figure 2C, relative bodyweight at 4 dpc, 8 dpc and 20 dpc increased by 10.3%, 19.3% and 37.7% for the G1 pigs, by 4.1%, 10.6% and 23.5% for the G2 pigs, by 9.0%, 18.5% and 36.2% for the G4 pigs, and by 10.4%, 21.2% and 39.8% for the G5 pigs, respectively (Figure 2C). Relative bodyweight of the G3 pigs had decreased by -8.0% at 4 dpc (Figure 2C). Compared to other groups, G2 pigs showed reduced relative bodyweight at 6 dpc and 8 dpc and G3 pigs at 4 dpc (*p* < 0.05).

### Antibody detection

**Figure 3:**
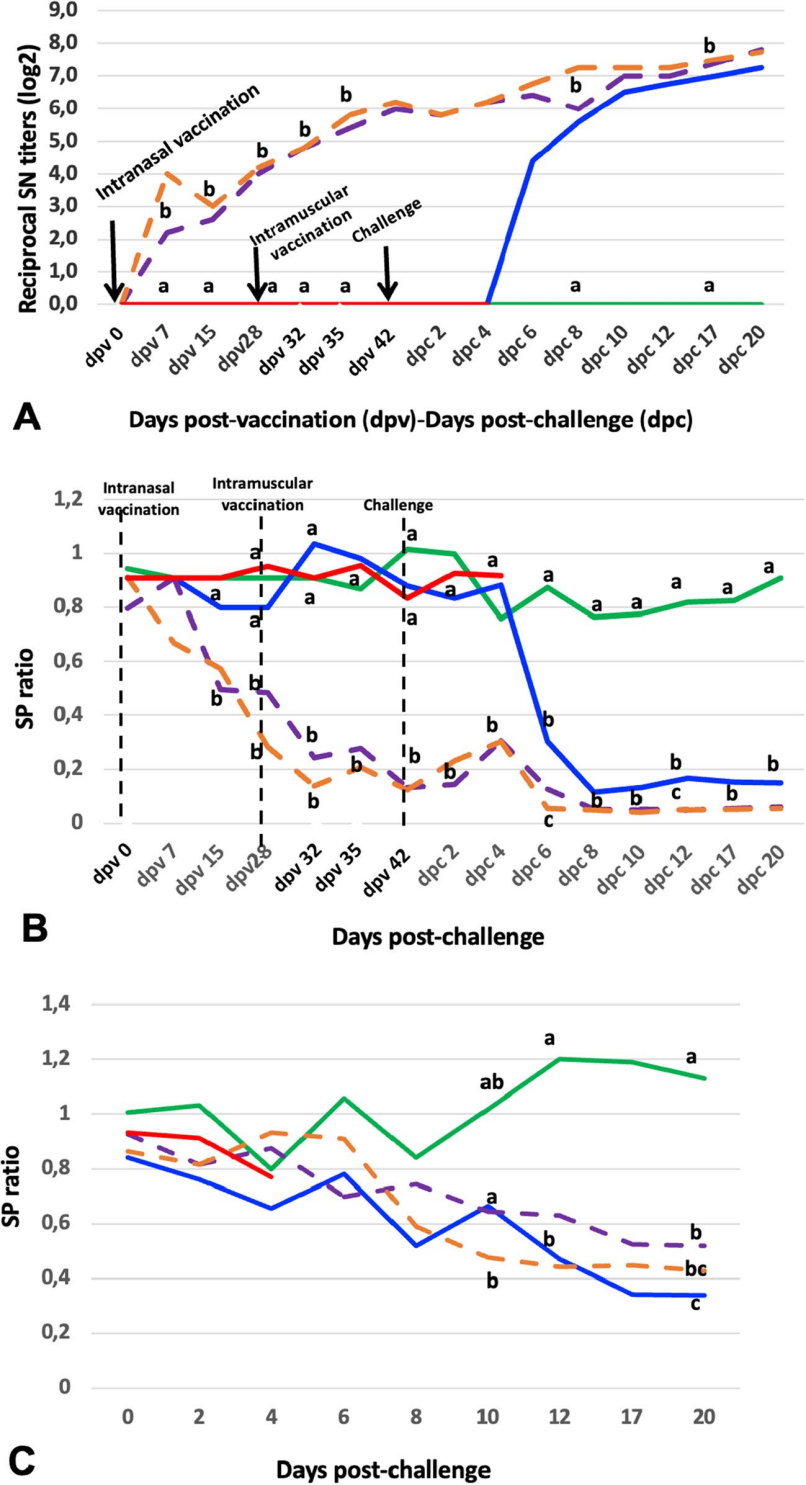
**Serology of unvaccinated or Bartha-K61 vaccinated pigs challenged with Hercules and HeN1 strains of SuHV1.** **A** Serum neutralization (SN), **B** gB-ELISA titres, **C**. gE-ELISA titres. G1-Unvaccinated and unchallenged control pigs (green line; n = 4); G2-Unvaccinated and Hercules-challenged pigs (blue line; *n* = 5); G3-Unvaccinated and HeN1-challenged pigs (red line; *n* = 5); G4-Vaccinated and Hercules-challenged pigs (dashed violet line; *n* = 5); G5-Vaccinated and HeN1-challenged pigs (dashed orange line; *n* = 5). Different letters at the same day post-challenge show significant difference (*p* < 0.05).

As expected SuHV1-gB, SuHV1-gE and seroneutralizing antibodies were not detected in the G1 control pigs at any time, nor in any of the G2 and G3 pigs prior to challenge (Figures 3A, 3B, 3C).

Neutralizing antibodies were detected in all pigs of G2 group at 6 dpc at titres ranging from 1/8 to 1/32 in a gradually increasing pattern until 20 dpc in the survivors (Figure 3A). No antibody was detected in G3 pigs at any time, though none survived beyond 4 dpc. SuHV1-gB antibodies were detected in all the surviving G2 pigs from 6 to 20 dpc, with levels reaching a plateau at 8 dpc to the end (Figure 3B). The development of SuHV1-gE antibodies in group G2 followed roughly a similar pattern as that of gB (Figure 3C).

In the vaccinated groups (G4 and G5), neutralizing antibodies were detected in all pigs at 10 doa e.g. seven days after the first (intranasal) vaccination, at titres ranging from 1/4 to 1/32. At 31 doa, e.g. day of booster (intramuscular) vaccination, all pigs had neutralizing antibody titres of 1/16 (Figure 3A). At 45 doa, e.g. date of challenge, all pigs (except one) had neutralizing titres of 1/64 or more, which gradually increased until 20 dpc.

In the vaccinated groups (G4 and G5), SuHV1-gB antibodies were detected in one only pig one week after primary vaccination (at 10 doa) and in 60% of the pigs at 18 doa. At 31 doa, e.g. day of booster (intramuscular) vaccination, almost all pigs had SuHV1-gB antibodies that remained until 20 dpc. Titres increased one week after the first intranasal vaccination up to the second intramuscular vaccination, and after a slight increase they reached a plateau up to the end of the trial (Figure 3B).

In the vaccinated groups (G4 and G5), SuHV1-gE antibodies were first detected in a few pigs at 2 dpc and 6 dpc. Sixty per cent (60%) of the pigs became gE positive at 8 dpc. A slight increase was observed over time, reaching a plateau at the end of the trial (Figure 3C).

### Virus excretion

No virus was isolated from nasal swabs of the G1 control pigs at any day (Table 2). All G2 and G3 pigs had excreted virus already at 2 dpc at doses higher than 5.2 log_10_ TCID_50_/g up to 8 dpc or until their euthanasia or death, if sooner. No virus was detectable in nasal excretions from unvaccinated pigs challenged with either strain after 8 days (Table 2).

**Table 2:** **Virus shedding in nasal excretions of 45-day old pigs non-vaccinated or vaccinated with the Bartha strain of SuHV1 and subsequently challenged with the Hercules or HeN1 strain of SuHV1**.

	Average virus titre^1^ at									
	0 dpc^1^	2 dpc	4 dpc	6 dpc	8 dpc	10 dpc	12 dpc	14 dpc	17 dpc	20 dpc
Group										
G1-control	0.0 (0/4)	0.0 (0/4)	0.0 (0/4)	0.0 (0/4)	0.0 (0/4)	0.0 (0/4)	0.0 (0/4)	0.0 (0/4)	0.0 (0/4)	0.0 (0/4)
G2-Hercules	0.0 (0/5)	5.2^a^ (5/5)	7.3^a^ (5/5)	4.5^a^ (5/5)	3.1^a^ (4/5)	0.0 (0/4)	0.0 (0/4)	0.0 (0/4)	0.0 (0/4)	0.0 (0/4)
G3-HeN1	0.0 (0/5)	6.6^a^ (5/5)	7.1^a^ (5/5)	–	–	–	–	–	–	–
G4-VacHercules	0.0 (0/5)	3.0^b^ (1/5)	3.3^b^ (5/5)	5.1^b^ (1/5)	0.0^b^ (0/5)	0.0 (0/5)	0.0 (0/5)	0.0 (0/5)	0.0 (0/5)	0.0 (0/5)
G5-VacHeN1	0.0 (0/5)	3.5^b^ (4/5)	4.5^b^ (5/5)	2.6^b^ (1/5)	0.0^b^ (0/5)	0.0 (0/5)	0.0 (0/5)	0.0 (0/5)	0.0 (0/5)	(0/5)

^1^log_10_/g tissue (shedding pigs/total alive pigs)

^2^dpc = days post-challenge.

Different letters at the same day post-challenge show significant difference (*p* < 0.05).

In most of the Hercules-challenged vaccinated pigs (G4), virus excretion was largely restricted to 4 dpc (Table 2), while in the majority of HeN1-challenged vaccinated pigs (G5) virus excretion occurred in most animals at both 2 and 4 dpc (Table 2). No virus was detected after 6 dpc in the nasal swabs in any challenged vaccinated pigs.

Fecal excretion of either strain was never detected in any pig at any time.

### Virus replication in tissues

No infectious virus was detected in the tissues of the G1 control pigs at any time.

In the unvaccinated pigs challenged with either strain (G2 and G3) and which had been euthanased or had died, virus was isolated from both the nasal mucosa and the brain at moderate titres. From three out of four Hercules-challenged pigs (G2) that survived until 20 doa, virus was recovered from the nasal mucosa of three pigs and the brain of only one pig at low levels (Table 1).

All vaccinated pigs survived and in only three G4 pigs and in two G5 pigs was virus recovered from nasal mucosa at low levels, and no virus was isolated from the brain of vaccinated pigs.

### Power analysis

Post-hoc power analyses using BW and body temperature data at 4 dpc e.g. when all pigs were alive have shown effect size 0.8897409 and power 0.8432532 for BW, and effect size 1.7227199 and power 0.9999979 for body temperature.

## Discussion

In the present study we compared the pathogenicity of two recently isolated field strains, one of European and the other of Chinese origin, on seronegative pigs and on Bartha-K61 vaccinated pigs.

Compared to farm studies, our containment system offered the opportunity to quantitatively assess the clinical effects of challenge or/and vaccine efficacy, with no other primary or secondary factors present that could influence the results during the trial. Indeed, though several researchers had previously performed field observations, leading to doubts over the efficacy of Bartha-K61 vaccines to protect pigs against SuHV1 field variants [15–18], the influence of possible co-infections or persistent infections with other farm pathogens (e.g. PRRSV, CSFV, PCV2, PPV, Mycoplasma suis etc.), the use of poor quality vaccines or storage, improper immunization schemes or/and the poor biosecurity practices could not be totally excluded [20, 27]. Therefore, firm conclusions on cross-genotype vaccine efficacy can only be determined by experimental studies in laboratory containment, where all confounding parameters can be efficiently controlled or excluded [28].

In contrast to studies using the pen as an experimental unit [21, 29–31], we used individually housed animals in this study. This way, the number of experimental animals was minimised, following the 3Rs principles (replacement, reduction, refinement), while simultaneously the statistical power of the results of the trial was increased. Indeed, the results indicated that in all cases, the achieved statistical power for the detection of the treatment effect was higher than the acceptable for a mean comparison statistical test, which is 80%.

The use of negative pressure-HEPA filtered kiosks, as well as preventing virus escape to the environment, ensured the absence of cross-contamination between animals, as evidenced by control animals remaining negative throughout the trial. Within the same group, the absence of cross-contamination may be beneficial as the health of less affected animals will not be worsened by the more affected ones, thus affecting their measurements.

When comparing the clinical effects of both strains in the seronegative pigs, a significant difference in their virulence was clear. Virulence of HeN1 strain was greater, compared to that of Hercules strain, as demonstrated by increased mortality, a shorter time to euthanasia or death and the higher clinical score (*p* < 0.05). It was notable that most HeN1 infected pigs succumbed without always showing severe neurological signs the previous day. Strains with increased virulence had already been demonstrated in previous studies employing European genotype strains [4, 5], but also in more recent studies employing Chinese genotype strains as well [16, 20, 29, 31]. In our study, HeN1 virus was isolated from the brain of all dead G3 seronegative pigs at titres ranging from 10^3.1^ to 10^5.6^ TCID_50_ /g tissue. However, we did not search at present for the exact sites of the brain or other organs that were affected, something that could give further information on the progression and fast course of the disease.

Past studies have been carried out on the efficacy of Bartha-K61-based vaccines against Chinese emerging isolates, using several different vaccine protocols and doses, animal models and ages, challenge routes and doses [15–21]. In other studies, pathogenicity had been assessed in non-porcine species (sheep, mice) as markers [12, 16, 29]. However, it is well-known for SuHV1 that viral neuropathogenesis in animal species which are not the natural host, may be different and that such extrapolation from non-porcine to porcine hosts may be misleading [32]. Also, skepticism on the appropriate vaccine doses, routes or/and schemes used in some studies have been expressed by several researchers [20, 27, 30, 33]. In our study, we have chosen to administer a very high challenge dose through the natural route on the host animal species, the pig, at a sensitive age, following the guidance in OIE Terrestrial Manual 2018 [28]. This model was much more severe than the challenge models of most of other studies where at least 10 or 100 times less virus had been administered in pigs of similar or older age [12, 15–17, 20, 21, 29–31, 33]. For conferring protection under such a severe challenge protocol, a vaccination scheme to induce both local and systemic immunity had been selected. From our results, it became clear that vaccination with Bartha-K61 vaccine could almost equally protect the pigs against both strains, as we demonstrated a dramatic reduction of morbidity and mortality, reduced and transient virus excretion and minimal or no pyrexia. It was also interesting that no virus was isolated from the brain of any of the vaccinated pigs in our study, while such was possible in one of the surviving Hercules-challenged non-vaccinated pigs at 20 dpc (Table 1), implying that establishment of latent infection is always a possibility which vaccination reduces efficiently.

Another critical parameter to evaluate a vaccine efficacy, of both health and economic importance, is bodyweight loss [4, 5, 28]. In our experiment both Hercules and HeN1 strain-challenged vaccinated pigs (G4 and G5) were not affected and followed exactly the same growth curve as the uninfected control pigs (Figure 2C) providing an additional evidence that the tested vaccine is efficacious against both strains.

Following infection of seronegative pigs by either strain, neutralizing antibodies were detected at 6 dpc, and at the same time by ELISA (gB at 4 dpc and gE at 6 dpc) with average neutralizing titres of 1/32 in moribund pigs. Intranasal vaccination also induced fast systemic immunity that gradually increased and was boosted by intramuscular vaccination. At the time of challenge, vaccinated pigs had an average neutralizing titre of 1/64. It should be remembered that protection or virus excretion are not related to the level of neutralizing antibodies but rather to cellular immunity [5, 21, 34]. Additional intranasal vaccination had established an important local immunity at the nasal mucosa, as evidenced by the reduced virus nasal replication (e.g. reduction of the number of excreting pigs and suppression of the length and quantity of excreted virus by both strains). Intranasal vaccination with Bartha-K61 strain induces better immunity (e.g. less severe clinical reaction) upon challenge compared to intramuscular vaccination [4]. Therefore, the vaccination scheme used at present (inducing both local and systemic immunity), as per the manufacturer’s protocol, is apparently more effective compared to a single or double intramuscular vaccination performed in most of the studies examining the efficacy of Bartha-K61 strain against Chinese novel strains [15–17, 21, 31], and may be a better model to use, especially when assessing cross-protection against diverse strains using very robust challenge models, such as is employed here.

Field isolates of SuHV1 –even within the same European genotype- may vary in their virulence [4, 5, 25]. During an outbreak in Northern Ireland, the NIA_3_ strain killed 13% of 14 to 20-week-old pigs while other field isolates such as NIA_1_ and NIA_2_ were not known to exhibit high virulence for pigs of this age [4–6]. The NIA_3_ strain was also shown to cause severe neurological signs in 15-week old pigs in experimental conditions [35]. However, when intensive eradication programmes based on gE deleted vaccines (e.g. Bartha-K61 strain) had been applied systematically, the wild-type virus was eradicated from such affected areas [36–38]. In contrast, if an eradication programme is not intensively and properly applied, e.g. as in Greece, it is expected that vaccination will simply suppress SuHV1 clinical signs, but will not eliminate the virus. This is why almost 30% of the Greek farms are gE-positive [11, 39]. Something similar may also apply in China e.g. in Shanghai area, where 67.8% of the pig farms are reported to be gE-positive [40].

The alternative vaccines tested so far in China are mostly derivatives of the wild type Chinese strains used for the respective challenges [15, 17, 18, 21, 25, 31]. Although it is anticipated that the more genetically related a vaccine is to the wild-type strain circulating in a region, the more complete may be its immunogenicity and thus protection, certain properties have to be tested over time for ensuring its success e.g. it has to be extensively studied for safety and efficacy, genetic stability, cross-protection against a wide range of field strains (provided that the infective strains in the field are essentially unknown).

Conclusively, this study had shown that Bartha-K61 vaccine strain, a universal long-term applied and tested vaccine, had been effective in protecting against novel emerging SuHV1 strains, if properly used.

## Abbreviations

SuHV1: Suid herpesvirus 1
AD: Aujeszky’s disease
doa: days of age
dpc: days post-challenge
AUT: Aristotle university of thessaloniki
PRRS: porcine reproductive and respiratory syndrome
PCV2: porcine circovirus 2
TCID_50_: tissue culture infectious doses
BW: bodyweight
PDCS: pig daily clinical score
SN: serum neutralization
PBS: phosphate buffered saline
ANOVA: analysis of variance
APHA: animal & plant health agency
CSFV: classical swine fever virus
PPV: porcine parvovirus
